# Supplementary material for: A long-term self-driven metronomic photodynamic system for cancer therapy
Source: Nat Commun. 2025 Oct 3;16:8823. doi: 10.1038/s41467-025-63868-3 (PMC12494783; doi:10.1038/s41467-025-63868-3)
Supplement: Supplementary file 1 — Supplementary Information [file 41467_2025_63868_MOESM1_ESM.pdf]

Supplementary Information of

A Long-term Self-driven Metronomic Photodynamic System for Cancer Therapy

Weili Wang[1,2,3,4,5,6#], Binglin Ye[1,2,3,4,5,6#], Yao Liu[1,2,3,4,5,6], Zhi Li[7], Qianying Huang[1,2,3,4,5,6] , Jialin Zhou[1,2,3,4,5,6], Min Hu[1,2,3,4,5,6], Jun Jiang[1,2,3], Weilin Wang[1,2,3,4,5,6*], Zhengwei Mao[1,2,3,4,5,6,8*], Yuan Ding[1,2,3,4,5,6*]

1 Department of Hepatobiliary and Pancreatic Surgery, The Second Affiliated Hospital, Zhejiang University School of Medicine, Zhejiang Hangzhou 310009, P. R. China.

2 Key Laboratory of Precision Diagnosis and Treatment for Hepatobiliary and Pancreatic tumour of Zhejiang Province, Zhejiang Hangzhou 310009, P. R. China

3 Research Center of Diagnosis and Treatment Technology for Hepatocellular Carcinoma of Zhejiang Province, Zhejiang Hangzhou 310009, P. R. China

4 Center for Medical Research and Innovation in Digestive System Tumors, Ministry of Education, Zhejiang Hangzhou 310009, China.

5 ZJU-Pujian Research & Development Center of Medical Artificial Intelligence for Hepatobiliary and Pancreatic Disease, Zhejiang Hangzhou 310058, P. R. China.

6 Cancer Center, Zhejiang University, Zhejiang Hangzhou 310058, P. R. China.

7 Department of Interventional Radiology, the First Affiliated Hospital of Soochow University, Soochow University, Jiangsu Suzhou 215001, China.

8 MOE Key Laboratory of Macromolecular Synthesis and Functionalization Department of Polymer Science and Engineering, Zhejiang University, Zhejiang Hangzhou 310027, China.

[#]These authors contributed equally *Correspondence to Dr. Yuan Ding Email: dingyuan@zju.edu.cn; Dr. Zhengwei Mao Email: zwmao@zju.edu.cn; Dr. Weilin Wang Email: wam@zju.edu.cn

Supplementary Table 1

Information for all using antibodies

Antibodies name	Catalog numbers	Supplier name	Dilutions	Clone numbers for monoclonals	Applicable /validation
Rabbit recombinant monoclonal Calreticulin antibody-conjugated toAlexa Flour 488	Cat. ab196158	Abcam	1:400	monoclonal	ICC/IF, Flow Cyt (Intra)
PerCP-Cy5.5 Hamster Anti-Mouse CD3e	Cat. 551163	BD Pharmingen	1:100	monoclonal	Flow Cyt
PE Anti-Mouse CD4	Cat. 100511	Biolegend	1:100	monoclonal	Flow Cyt
FITC Rat Anti-Mouse CD8a	Cat. 553030	BD Pharmingen	1:100	monoclonal	Flow Cyt
APC-Cy7 Rat Anti-Mouse CD45	Cat. 557659	BD Pharmingen	1:100	monoclonal	Flow Cyt
FITC anti-mouse CD11c Antibody	Cat. 117305	Biolegend	1:100	monoclonal	Flow Cyt
APC Hamster anti-Mouse CD80	Cat. 560016	BD Pharmingen	1:100	monoclonal	Flow Cyt
PE-Cy7 Rat Anti-Mouse CD86	Cat. 560582	BD Pharmingen	1:100	monoclonal	Flow Cyt
Alexa Fluor® 488-conjugated Goat Anti-Rabbit IgG (H+L)	Cat. GB25303	Servicebio	3:10	polyclonal	IF, FC
Cy3 conjugated Donkey Anti-Rabbit IgG (H+L)	Cat. GB21403	Servicebio	1:10	polyclonal	IF, FC
Recombinant Anti-CD3 antibody (Rabbit mAb)	Cat. GB150004	Servicebio	1:1000	monoclonal	IHC/IF
Recombinant Anti-CD4 antibody (Rabbit mAb)	Cat. GB15064	Servicebio	1:200	monoclonal	IHC/IF
Recombinant Anti-CD8 alpha antibody (Rabbit mAb)	Cat. GB15068	Servicebio	1:400	monoclonal	IHC/IF
Anti-CD86 antibody	Cat. ab270719	Abam	1:200	monoclonal	ICC/IF, IHC-P
Anti-Mannose Receptor/CD206 Rabbit pAb	Cat. GB113497	Servicebio	1:400	polyclonal	IHC/IF

Supplementary Table 2

Cell line identification by STR analysis

STR loci	A375 in our study		A375 in databases	
	**Allele1**	**Allele2**	**Allele1**	**Allele2**
Amelogenin	X		X	
CSF1PO	11	12	11	12
D2S1338	16	24	16	24
D3S1358	15	17	15	17
D5S818	12		12	
D7S820	9		9	
D8S1179	11	14	11	14
D13S317	11	14	11	14
D16S539	9		9	
D18S51	12	17	12	17
D19S433	13	14.2	13	14.2
D21S11	29	30	29	30
FGA	23		23	
Penta D	9	15	9	15
Penta E	10	12	10	12
TH01	8		8	
TPOX	8	10	8	10
vWA	16	17	16	17
D6S1043	11	14		
D12S391	18	21	18	21
D2S441	11		11	

Note: ExP ASy database was used for cell line comparison. The authentication was performed by Procell. System (Wuhan, China).

Supplementary Table 3

Cell line identification by STR analysis

STR loci	Hep3B in our study		Hep3B in databases	
	**Allele1**	**Allele2**	**Allele1**	**Allele2**
Amelogenin	X		X	
CSF1PO	8		8	
D2S1338	21	25	21	25
D3S1358	15		15	
D5S818	13		13	
D7S820	8	10	8	10
D8S1179	12		12	
D13S317	12	14	12	14
D16S539	10		10	
D18S51	20		20	
D19S433	12.2	14	12.2	14
D21S11	30	31	30	31
FGA	18		18	
Penta D	12	14	12	14
Penta E	5	16	5	16
TH01	6	7	6	7
TPOX	9		9	
vWA	17		17	
D6S1043	12	17		
D12S391	17			
D2S441	10	12		

Note: ExP ASy database was used for cell line comparison. The authentication was performed by Procell. System (Wuhan, China).

Supplementary Table 4

Cell line identification by STR analysis

STR loci	B16 in our study			B16 in databases		
	**Allele1**	**Allele2**	**Allele3**	**Allele1**	**Allele2**	**Allele3**
1-1	17	18		17	18	
1-2	19	20		19	20	
2-1	16			16		
3-2	14	15		14	15	
4-2	20.3	21.3		20.3	21.3	
5-5	16	20		16	20	
6-4	18	19		18	19	
6-7	15	16		15	16	
7-1	26.2			26.2		
8-1	16	17		16	17	
11-2	16	17		16	17	
12-1	17	18		17	18	
13-1	17.1	18.1		17.1	18.1	
15-3	22.3	23.3		22.3	23.3	
17-2	15	16	17	15	16	
18-3	15	16		15	16	
19-2	13			13		
X-1	28			28		
TH01						
D5S818						

Note: CCRID database was used for cell line comparison. The authentication was performed by Procell. System (Wuhan, China).

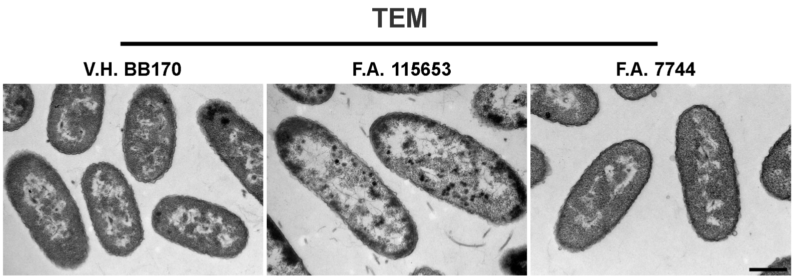

Supplementary Fig. 1 Morphology of luminous bacteria

The bioluminescent bacteria were collected by centrifugation at 100 × g for 10 mins in 15 mL Eppendorf. Then fixed by 2.5% glutaric dialdehyde solution at room temperature in the dark for 30 minutes, An ultra-thin copper 60 film to visualize their morphology by HR-TEM (Hitachi TEM system) at 80 kV, Scale bar: 500 nm.

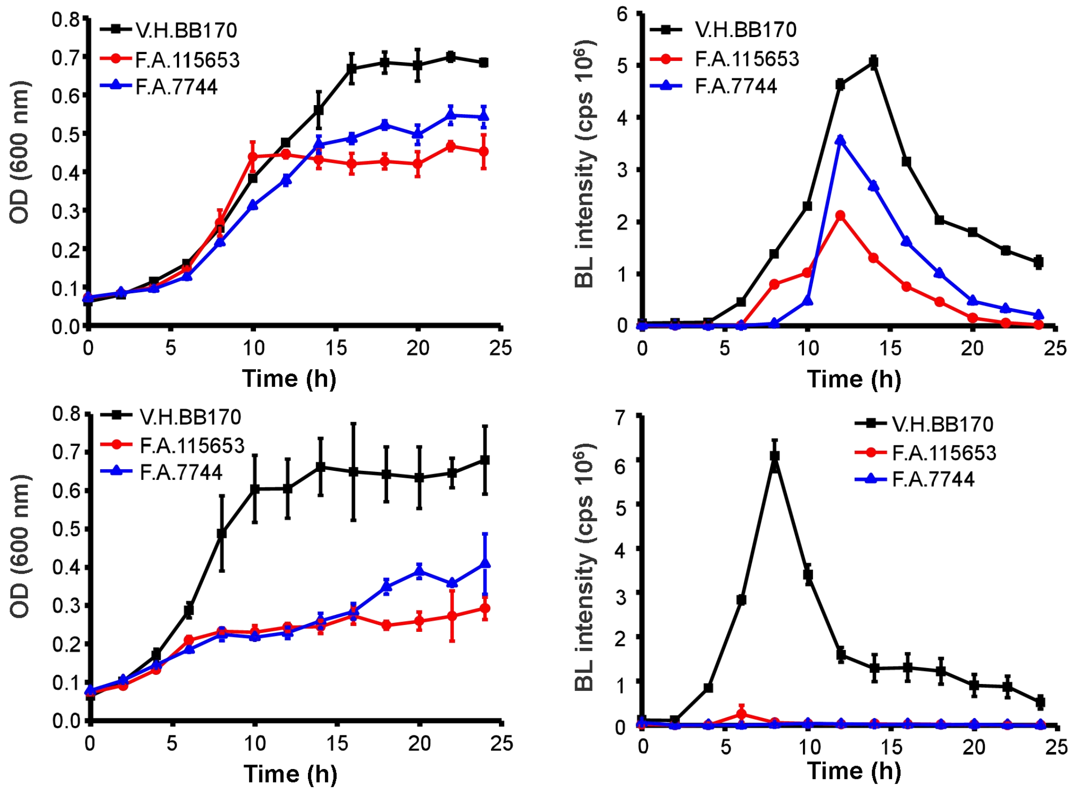

Supplementary Fig. 2 Growth curves optical density (OD) and Bioluminescence (BL) intensity of bioluminescent bacteria in the presence of 25 °C (up) and 37 °C (down) by recording the bioluminescence intensity and optical absorbance of incubation at 600 nm (n =3 independent experiments). The data are presented as mean ± SD.

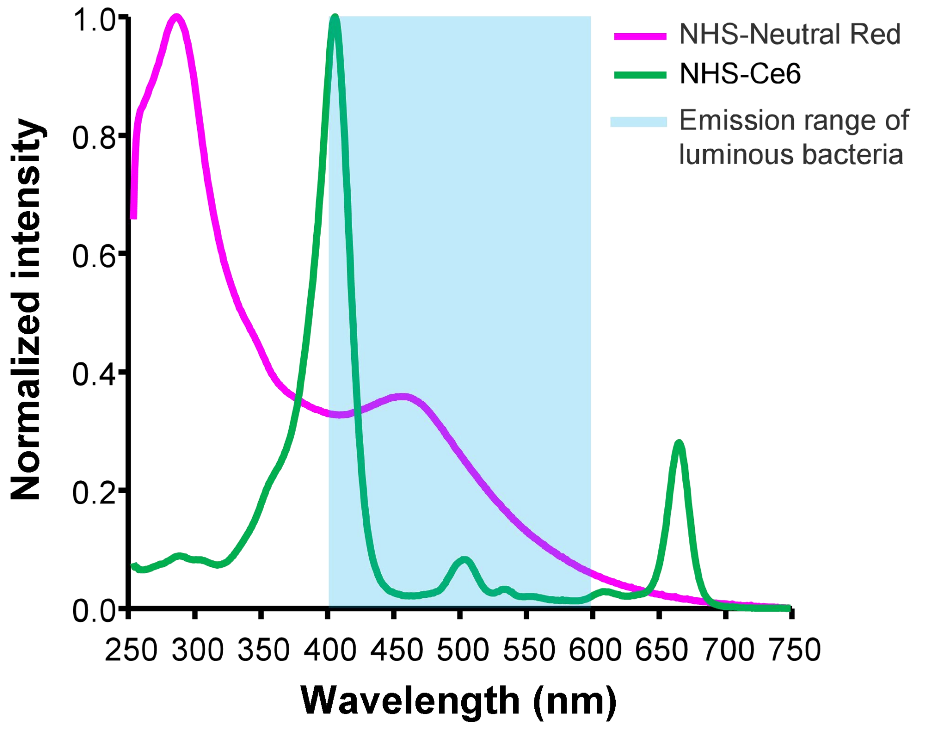

Supplementary Fig. 3 Absorption spectra of photosensitizers

NHS-Neutral red and NHS-Ce6 were used to examine their absorption spectrum by UV-Vis spectrophotometer. (Thermo Fisher Scientific Evolution 350 from 250-750 nm).

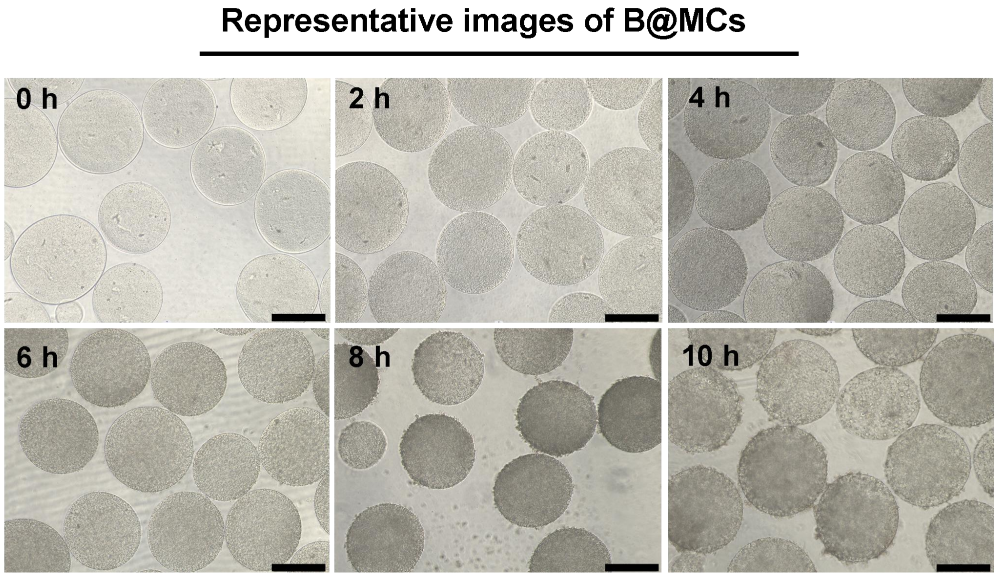

Supplementary Fig. 4 Representative images of B@MCs

The B@MCs were cultured in 2216E media at 3600/mL 37 °C to capture images at 0, 2, 4, 6, 8 and 10 h. Scale bars: 100 μm.

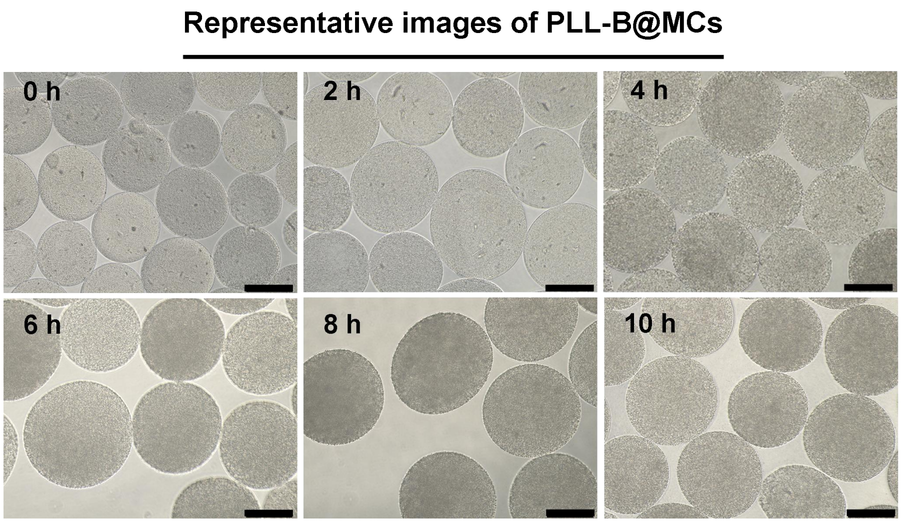

Supplementary Fig. 5 Representative images of PLL-B@MCs

The PLL-B@MCs were cultured in 2216E media at 3600/mL 37 ℃ to capture images at 0, 2, 4, 6, 8 and 10 h. Scale bars: 100 μm.

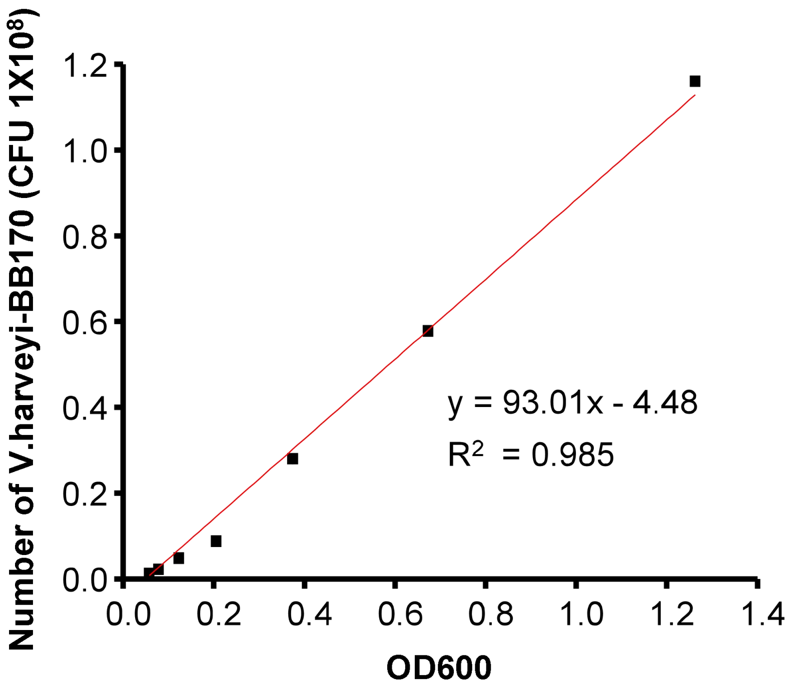

Supplementary Fig. 6 Optical density (OD600) v.s. Number of V.H.BB170 (CFU $1{\times}10^8$) in the presence of 37 °C in 2216E agar medium by recording the colony forming unit.

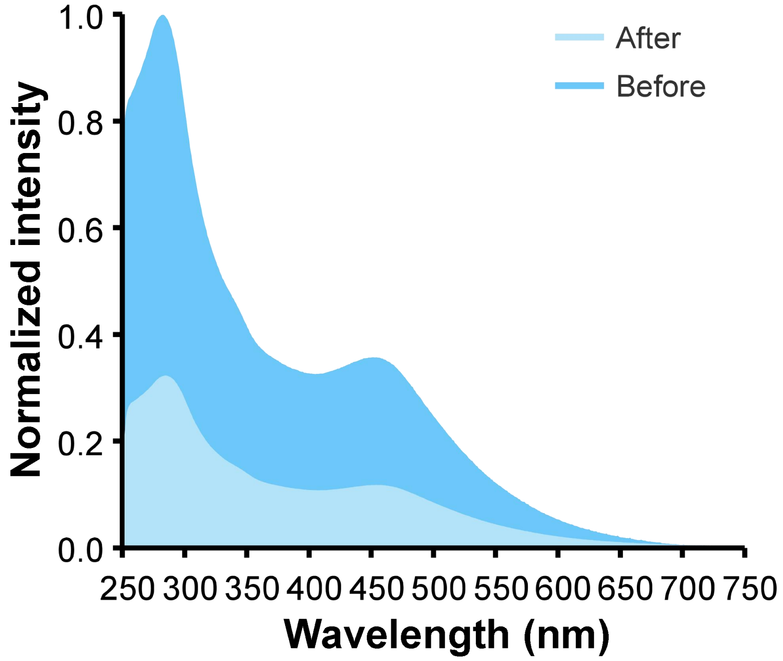

Supplementary Fig.7 Photosensitizer binding efficiencies test

500 µL, 2 mg/mL NHS-NR were dispersed in PLL-B@MCs (500 µL, 3.6 × 10^4/mL)) for 20 mins for reaction. Before and after reaction, NHS-NR were examined their absorption spectrum by UV-Vis spectrophotometer. (Thermo Fisher Scientific Evolution 350 from 250-750 nm).

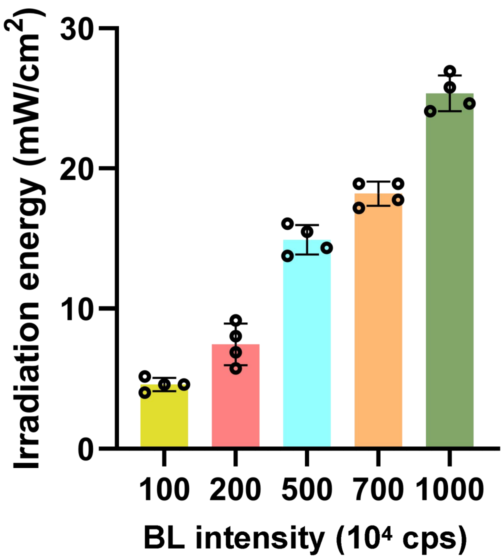

Supplementary Fig. 8 Correlation analysis of BL intensity (10^4 cps) versus irradiation energy (mW/cm^2) for PB@MCs, recorded with a VLP-2000 laser power meter (n = 4 independent experiments). Error bars, mean ± SD.

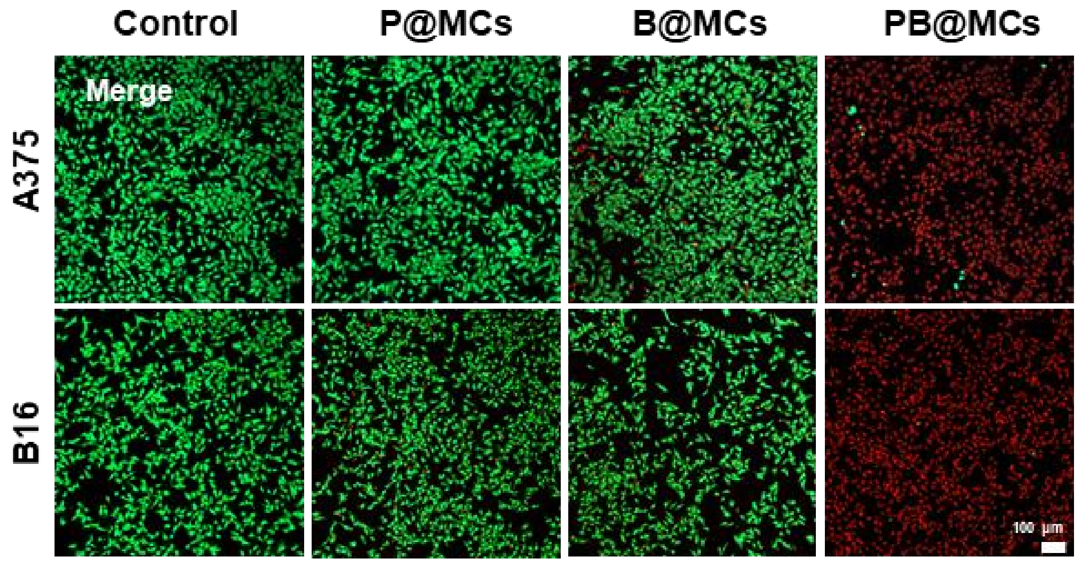

Supplementary Fig. 9 Fluorescence microscopy images following LIVE/DEAD staining *in vitro*. A375 and B16 cells were treated with 200 µL of either PBS, Empty MCs linked to photosensitizer (P@MCs), MCs encapsulating BB170 bacteria (B@MCs), or PB@MCs for 8 hours, followed by staining with a LIVE/DEAD kit for microscopy visualization. Red fluorescence represents dead cells and green fluorescence represents live cells. Scale bars: 100 µm.

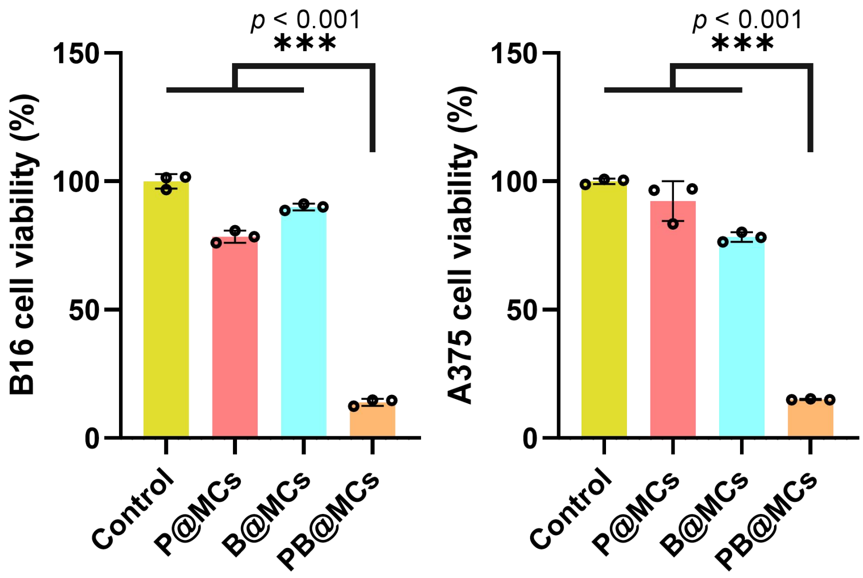

Supplementary Fig. 10 Cell viability assessment *in vitro*. B16 and A375 cells were exposed to 200 μL of P@MCs, B@MCs, or PB@MCs in dark conditions for 8 hours. Viabilities were determined using an CCK-8 assay (n = 3 independent experiments). The data are presented as mean ± SD. Statistical significance is noted with ****p < 0.001, compared to the data for P@MCs, B@MCs and Control group according to one-way ANOVA test combination with Tukey's multiple comparisons by GraphPad Prism 9 XML project software.

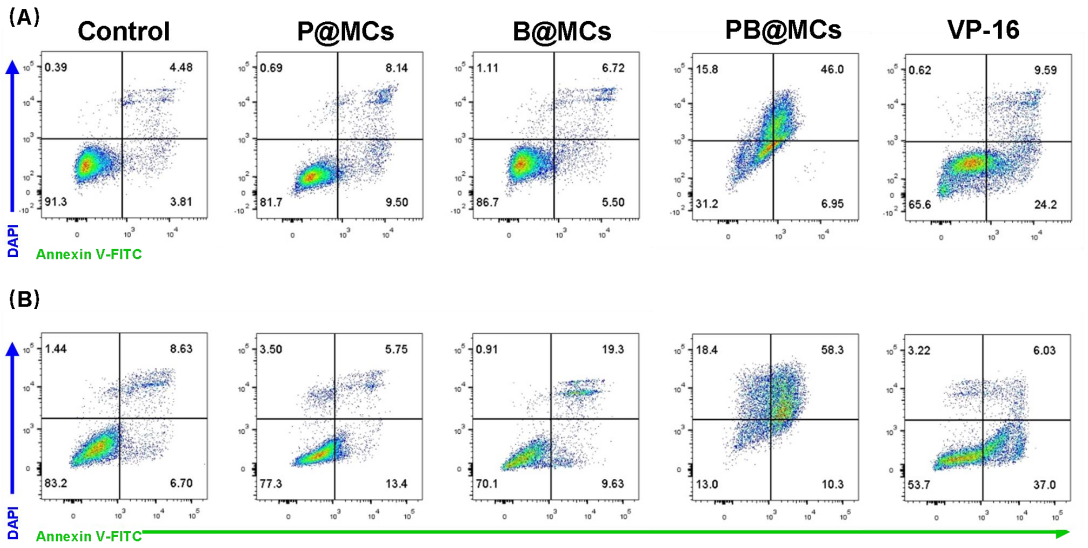

Supplementary Fig. 11 Representative images of cells by flow cytometry analysis *in vitro*.

(A) A375 and (B) Hep3B cells seeded in 6-transwell plates (5×10^5 cells/well) were exposed to bare B@MCs, P@MCs, and PB@MCs at 400 μL or 400 μg/mL VP-16. After 8 h incubation, the cells were stained by Annexin V-FITC and PI for flow cytometry analysis.

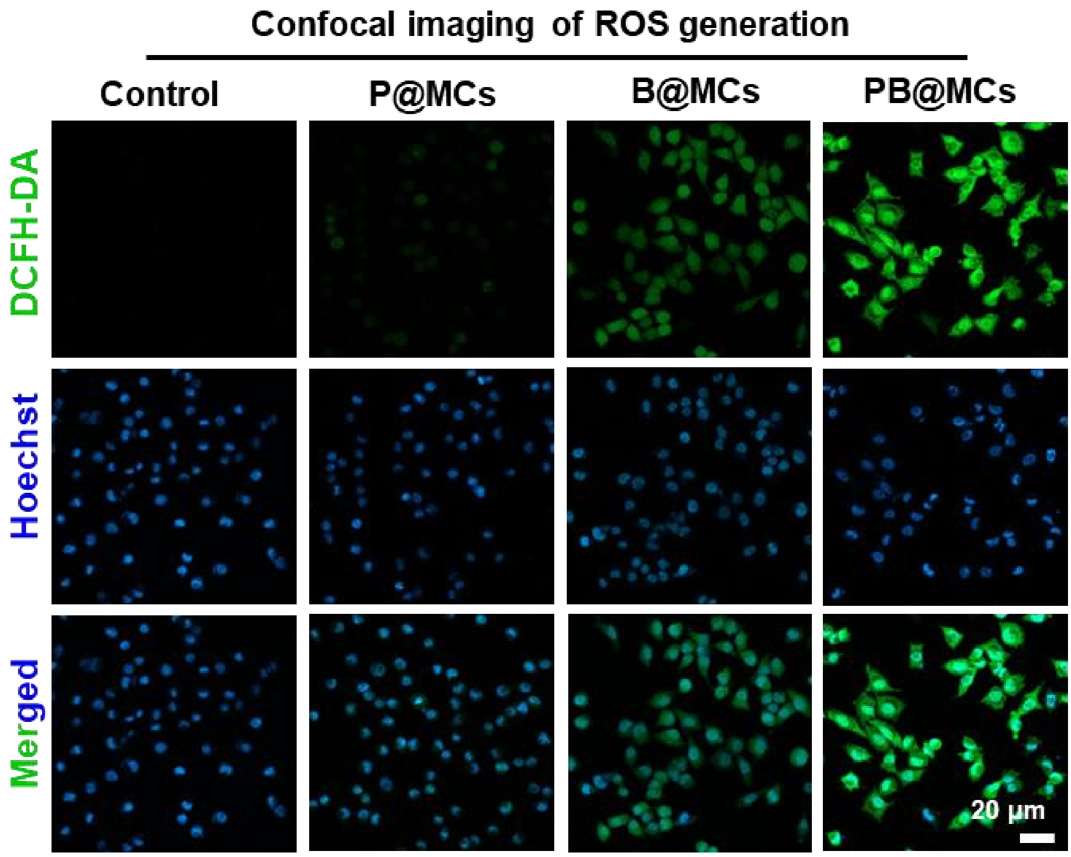

Supplementary Fig. 12 Confocal images of A375 cells by the probe of DCFH-DA *in vitro*

Confocal microscopy of A375 cells using DCFH-DA probe. A375 cells, seeded in 24-transwell plates at 5×10^4 cells/well, were treated with P@MCs, B@MCs, and PB@MCs and subsequently stained with the ROS-specific probe DCFH-DA after 2 h. Green: DCF, Blue: Hoechst.

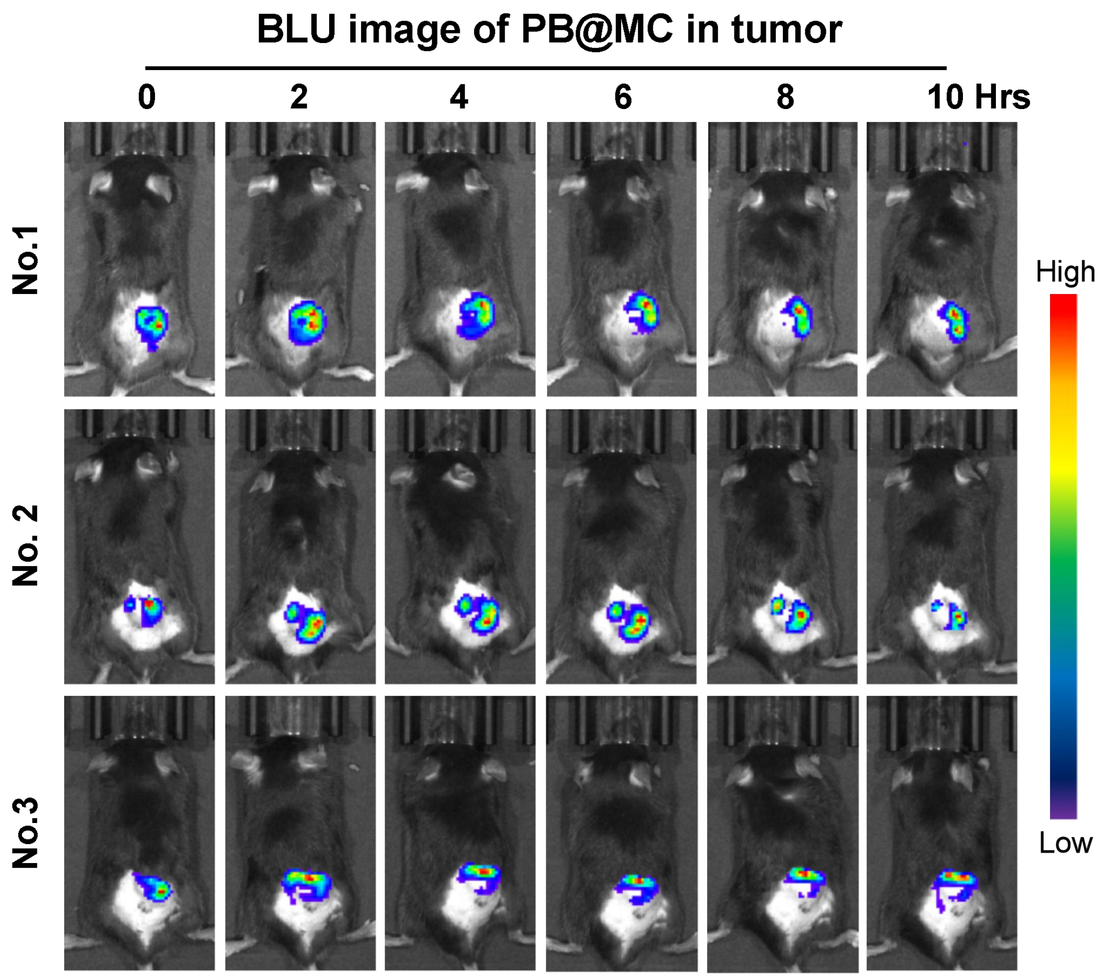

Supplementary Fig. 13 Bioluminescence image (BLI) of B16 melanoma-bearing mice injected with PB@MCs.

B16 melanoma-bearing mice with tumour volumes ~300 mm^3 were intratumourally injected with 50 μL of PB@MCs. Bioluminescence imaging was performed using an IVIS imaging system (PerkinElmer, IVIS Lumina Series III) at 0, 2, 4, 6, 8, and 10 hours post-injection (n = 3 mice). All pictures were analysis by Living image 4.8.0 software.

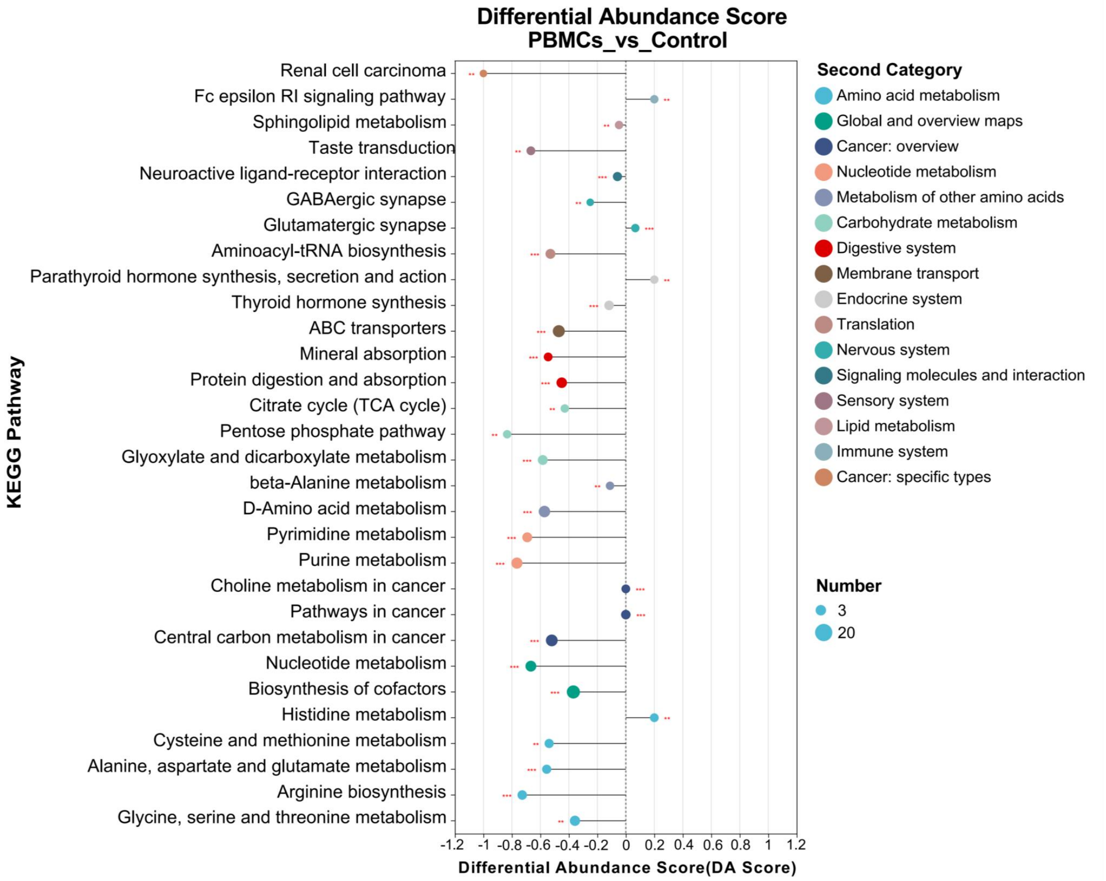

Supplementary Fig. 14 Differential abundance score of metabolites.The horizontal lines on the left and right sides of the central axis show significant decrease and increase in the level of metabolites in the pathway. The size of dots illustrates the number of differential metabolites in each pathway.

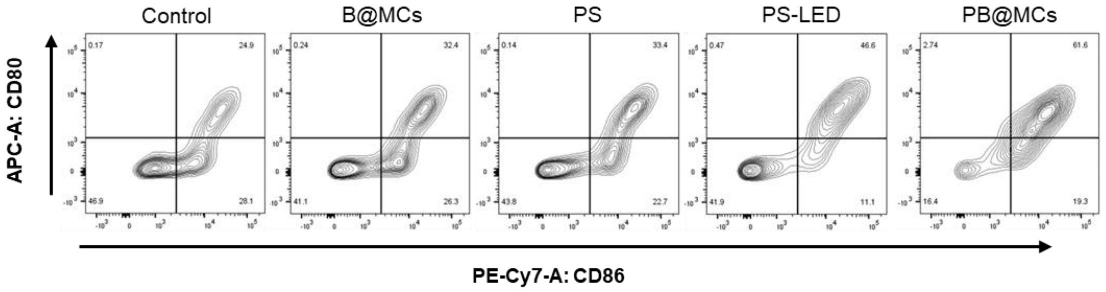

Supplementary Fig. 15 Percentage of DC maturation ($CD80^{+}CD86^{+}$ DCs) after exposure to different treatments in B16 cells.

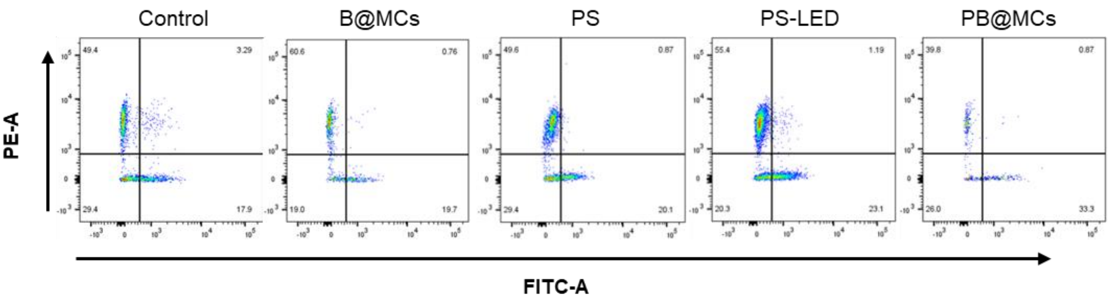

Supplementary Fig. 16 Quantitative analysis of $CD8^{+}$ T cell intratumoural infiltration after exposure to different treatments in B16 tumour tissues.

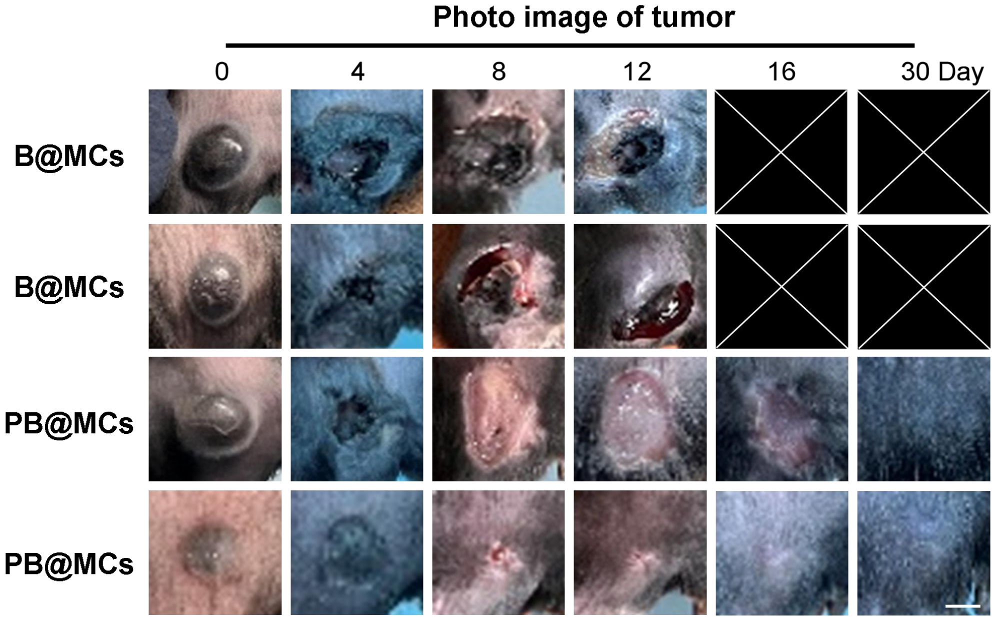

Supplementary Fig. 17 Optical photographs of tumour-bearing mice under B@MCs and PB@MCs treatments for 30 day (n = 2 mice). Scale bar: 5 mm.

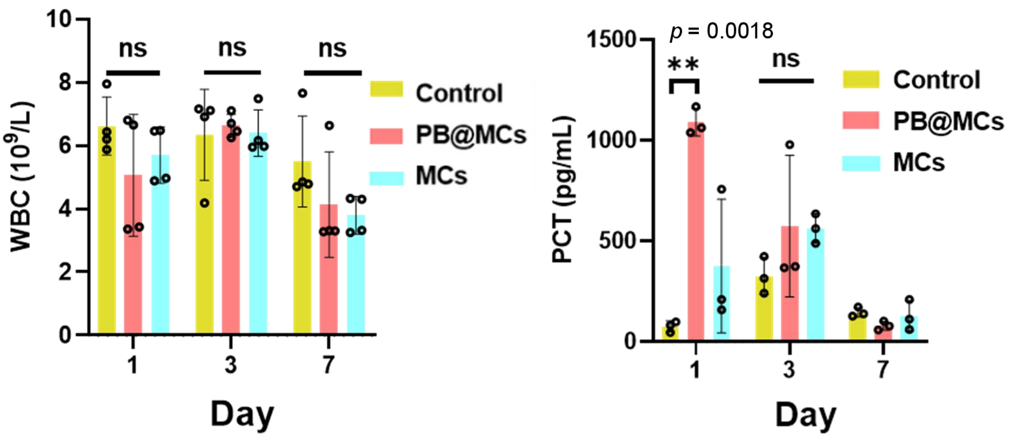

Supplementary Fig. 18 Blood test of WBC and PCT in healthy mice

Blood was collected from 8-week-old C57B6 mice by eyeball blood collection on day 1, 3 and 7 after treatment, including injection of 50 μL PBS, PB@MCs, and MCs.(n =4 independent experiments for WBC and n =3 independent experiments for PCT). Error bars, mean ± SD. Statistical significance is noted with $**p < 0.01$, compared to the data for PBS, PB@MCs, and MCs group according to one-way ANOVA test combination with Tukey's multiple comparisons by GraphPad Prism 9 XML project software, ns: not significant.

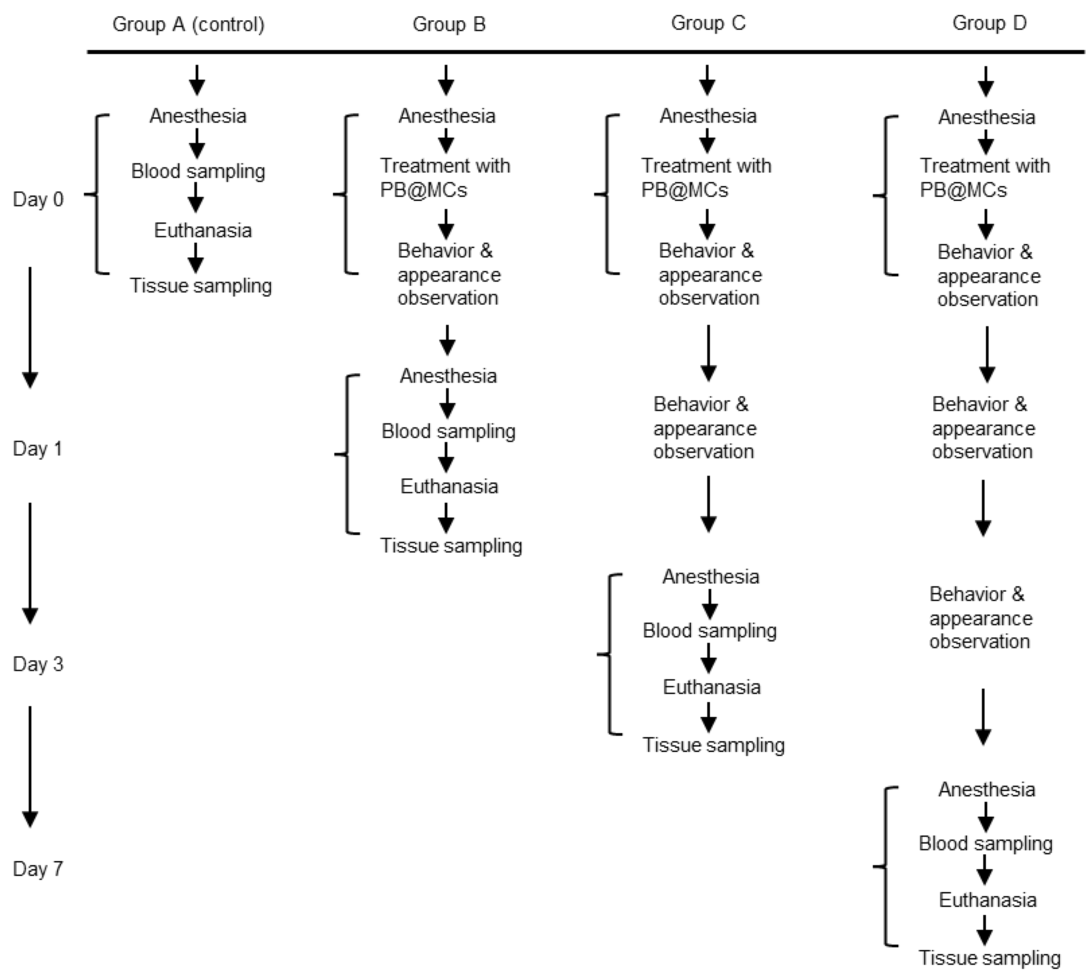

Supplementary Fig. 19 Flowchart and timeline of the experimental design. Group A (control): non-treated mice undergo anesthesia and blood sampling before euthanasia for tissue sampling on Day 0. Groups B to D: mice treated with PB@MCs (30 μL) on Day 0 undergo all procedures (*i.e.*, anesthesia, blood sampling, euthanasia and tissue sampling) on Day 1 (Group B), Day 3 (Group C) or Day 7 (Group D). The mouse appearance and behavior are monitored throughout the experiment.

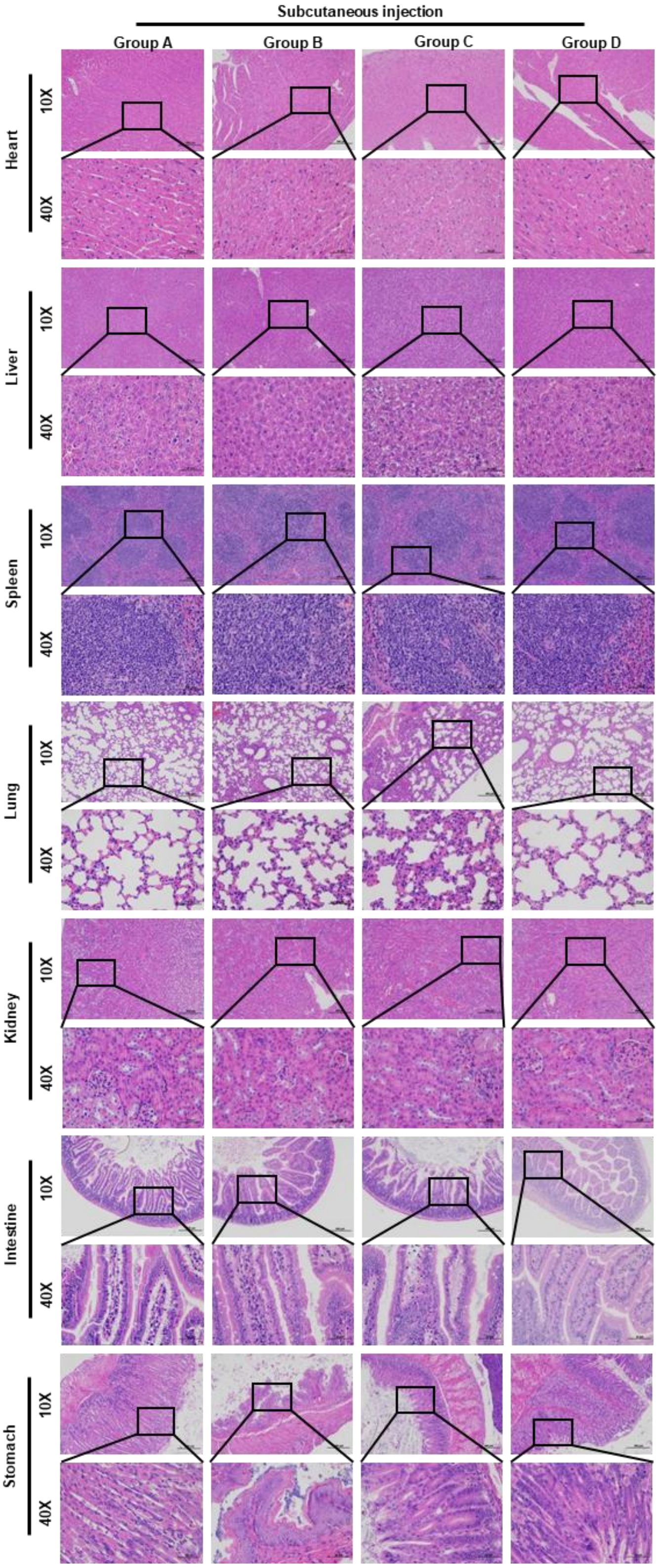

Supplementary Fig. 20 Representative histological H&E staining images of organs in mice H&E staining for subcutaneous microscopy imaging after 1st, 3rd and 7th d after injection of 30 μL PB@MCs. The animals were sacrificed to collect heart, liver, spleen, lung, kidney, stomach and intestine. The tissue samples were fixed in 10% formalin for H&E staining. Scale bar: 200 μm for 10×, and 50 μm for 40×.

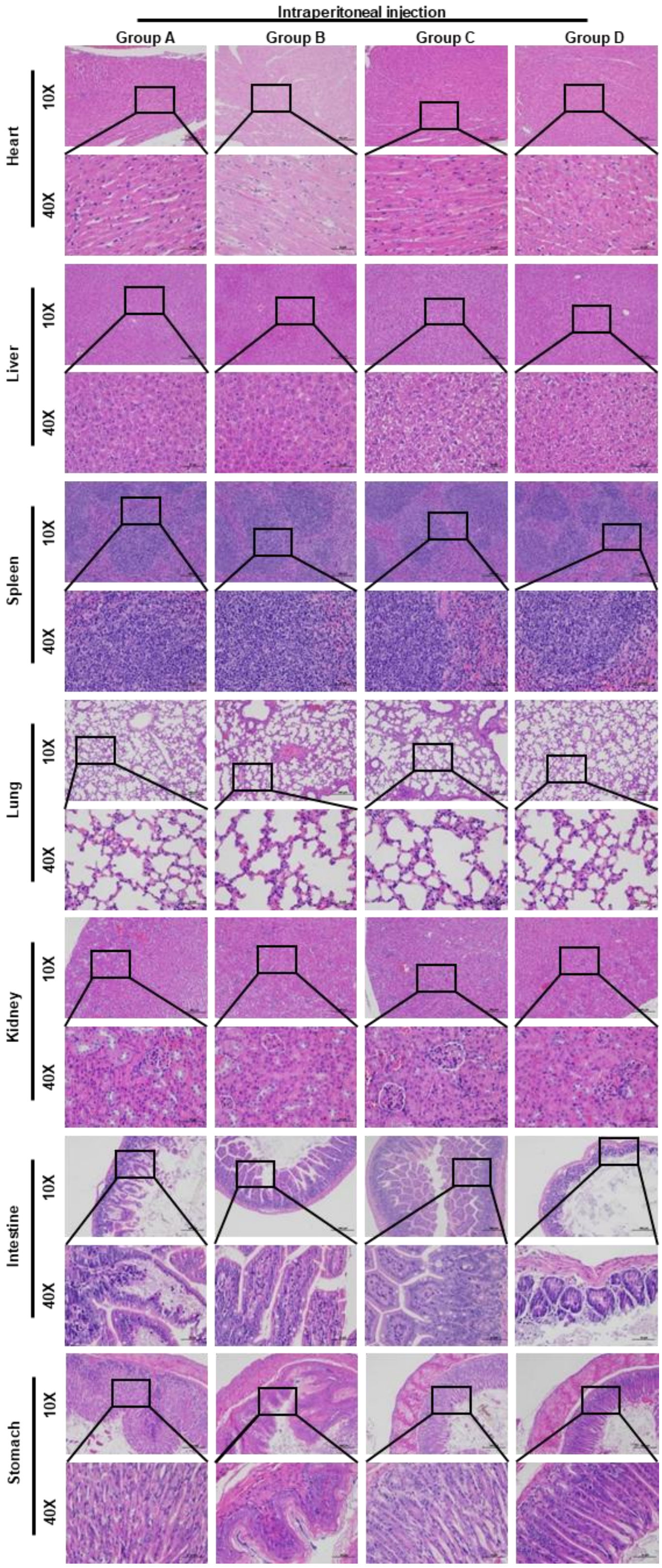

Supplementary Fig. 21 Representative histological H&E staining images of organs in mice H&E staining for intraperitoneal microscopy imaging after 1st, 3rd and 7th d after injection of 30 μL PB@MCs. The animals were sacrificed to collect heart, liver, spleen, lung, kidney, stomach and intestine. The tissue samples were fixed in 10% formalin for H&E staining. Scale bar: 200 μm for 10×, and 50 μm for 40×.

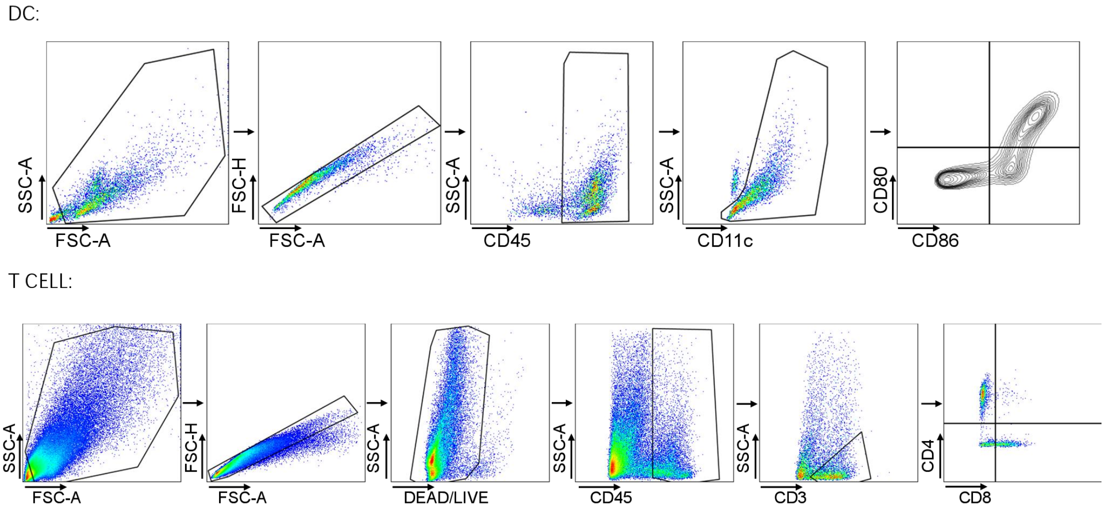

Supplementary Fig. 22 Flow cytometry gating strategy for the analysis of DC in vitro and T cells in the tumour.
