# Supplementary material for: A long-term self-driven metronomic photodynamic system for cancer therapy
Source: Nat Commun. 2025 Oct 3;16:8823. doi: 10.1038/s41467-025-63868-3 (PMC12494783; doi:10.1038/s41467-025-63868-3)
Supplement: Supplementary file 2 — Reporting Summary [file 41467_2025_63868_MOESM2_ESM.pdf]

nature portfolio

Corresponding author(s): Zhengwei Mao

Last updated by author(s): Aug 12, 2025

# Reporting Summary

Nature Portfolio wishes to improve the reproducibility of the work that we publish. This form provides structure for consistency and transparency in reporting. For further information on Nature Portfolio policies, see our Editorial Policies and the Editorial Policy Checklist.

## Statistics

For all statistical analyses, confirm that the following items are present in the figure legend, table legend, main text, or Methods section.

| n/a | Confirmed | |
|---|---|---|
| ☐ | ☒ | The exact sample size (*n*) for each experimental group/condition, given as a discrete number and unit of measurement |
| ☐ | ☒ | A statement on whether measurements were taken from distinct samples or whether the same sample was measured repeatedly |
| ☐ | ☒ | The statistical test(s) used AND whether they are one- or two-sided<br>*Only common tests should be described solely by name; describe more complex techniques in the Methods section.* |
| ☒ | ☐ | A description of all covariates tested |
| ☒ | ☐ | A description of any assumptions or corrections, such as tests of normality and adjustment for multiple comparisons |
| ☐ | ☒ | A full description of the statistical parameters including central tendency (e.g. means) or other basic estimates (e.g. regression coefficient) AND variation (e.g. standard deviation) or associated estimates of uncertainty (e.g. confidence intervals) |
| ☐ | ☒ | For null hypothesis testing, the test statistic (e.g. *F*, *t*, *r*) with confidence intervals, effect sizes, degrees of freedom and *P* value noted<br>*Give P values as exact values whenever suitable.* |
| ☒ | ☐ | For Bayesian analysis, information on the choice of priors and Markov chain Monte Carlo settings |
| ☒ | ☐ | For hierarchical and complex designs, identification of the appropriate level for tests and full reporting of outcomes |
| ☒ | ☐ | Estimates of effect sizes (e.g. Cohen's *d*, Pearson's *r*), indicating how they were calculated |

*Our web collection on statistics for biologists contains articles on many of the points above.*

## Software and code

Policy information about availability of computer code

Data collection

1. The morphology and size of MCs, P@MCs, B@MCs and PB@MCs were determined by microscopy (FV1200, Olympus, Tokyo, Japan).
2. Long-term Bioluminescence intensity of PB@MCs were measured by a Microplate reader (Tecan spark, Switzerland).
3. Bioluminescence spectra of B@MCs and PB@MCs were measured with an Edingbour NanoSpectralyzer fluorimetric analyzer (Applied NanoFluorescence, FLS980).
4. The energy radiation density of PB@MCs were recorded using a Laser Power Meter (YanbangTech VLP2000, Beijing, China).
5. The cell viability data was collected by a Microplate reader (Tecan spark, Switzerland).
6. Live/Dead and ROS Cell imaging were visualized by confocal microscopy (LSM900 Carl Zeiss, Germany).
7. Flow cytometry was performed on FACSCanto II, BD Biosciences and data was acquired with the help of FlowJo software 10.6.2 (Tree Star Inc).
8. Bioluminscence imaging of Bacteria and PB@MCs were imaged by Xiaomi 14 smartphone(China) and Canon camera (Japan).
9. CT imaging of rabbits were collected by a 16-slice CT spiral scan (Brilliance-16, Phillips, USA) guidance.
10. The tumors were imaged by Canon camera (Japan).
11. Hematoxylin and eosin (H&E)staining images were acquired using a Digital Slide Scanner (HAMAMATSH PHOTONICS, NanoZoomerS360).

| Data analysis | 1. Flow cytometry data were analyzed by FlowJo software 10.6.2(Tree Star Inc).<br>2. Numerical data were statistically analyzed and graphs were generated with the help of Origin85 software and Microsoft excel software.<br>3. Raw data of animal imaging and bioluminescence imaging were analyzed with the help of Microsoft office software 2010, ImageJ 8.0 and Living imaging software version 4.5.2 (PerkinElmer).<br>4. Hematoxylin and eosin (H&E) staining imaging were visualized by NDP,view (Version 2.9.22).<br>5. Immunofluorescence images were visualized by CaseViewer (version 2.4) |
|---|---|

For manuscripts utilizing custom algorithms or software that are central to the research but not yet described in published literature, software must be made available to editors and reviewers. We strongly encourage code deposition in a community repository (e.g. GitHub). See the Nature Portfolio guidelines for submitting code & software for further information.

## Data

Policy information about availability of data

All manuscripts must include a data availability statement. This statement should provide the following information, where applicable:

- Accession codes, unique identifiers, or web links for publicly available datasets
- A description of any restrictions on data availability
- For clinical datasets or third party data, please ensure that the statement adheres to our policy

All data needed to support the findings of this study are provided within the article and Supplementary information.

## Research involving human participants, their data, or biological material

Policy information about studies with human participants or human data. See also policy information about sex, gender (identity/presentation), and sexual orientation and race, ethnicity and racism.

| Reporting on sex and gender | Female mice and rabbits was used for establishment of melanoma tumor bearing mice and hepatocarcinoma rabbit in this study |
|---|---|
| Reporting on race, ethnicity, or other socially relevant groupings | There are no human participants in this study |
| Population characteristics | It is not applicable for this study |
| Recruitment | It is not applicable |
| Ethics oversight | This study was approved by the Laboratory Animal Welfare and Ethics Committee of The Second Affiliated Hospital of Zhejiang University School of Medicine (ARIB-2023-1520) |

Note that full information on the approval of the study protocol must also be provided in the manuscript.

# Field-specific reporting

Please select the one below that is the best fit for your research. If you are not sure, read the appropriate sections before making your selection.

[x] Life sciences  [ ] Behavioural & social sciences  [ ] Ecological, evolutionary & environmental sciences

For a reference copy of the document with all sections, see nature.com/documents/nr-reporting-summary-flat.pdf

# Life sciences study design

All studies must disclose on these points even when the disclosure is negative.

| Sample size | Mead's resource equation was used determine the animal numbers in most experiments. Mouse and Rabbit numbers in survival rate experiment were determined by an equation for dichotomous variables (National Journal of Physiology, Pharmacy and Pharmacology 2011;1:35-39). The number of cell samples were determined on the preliminary test results as well as the minimal requirements in Student T-test. For property measurement experiments, samples were prepared and tested at least twice. For in vitro and in vivo studies, each group contains at least three independent experimnets. The number of the independent experiments was indicated in each figure legend and Methods section. |
|---|---|
| Data exclusions | No data was excluded from the analysis. |
| Replication | All the findings were reliably reproduced in multiple independent experiments. For property measurement experiments, our data represent at least two independent assays that produce similar results. We have used different assays to confirm our findings. For in vitro and in vivo studies, each group contains at least three independent experiments with similar results. |
| Randomization | All the cell and animal experiments were randomly allocated into experimnetal groups by throwing dice. |
| Blinding | Blind trals were conducted in several critical experiments, including impacts of PB@MCs on cancer cell proliferation, biosafy assessment ROS testing and animal experiments (e.g. In vivo and ex vivo images, DCF-DA assay, H&E staining, immunohistochemical staining, tumor growth |

curves and survival rate). Blinding was not available in some experiments and studies because the operator could easily identify the structure by characteristic signals (TEM, Microscopy images of bacteria, PB@MCs, emission spectra of PB@MCs) .

# Behavioural & social sciences study design

All studies must disclose on these points even when the disclosure is negative.

| | |
|---|---|
| Study description | *Briefly describe the study type including whether data are quantitative, qualitative, or mixed-methods (e.g. qualitative cross-sectional, quantitative experimental, mixed-methods case study).* |
| Research sample | *State the research sample (e.g. Harvard university undergraduates, villagers in rural India) and provide relevant demographic information (e.g. age, sex) and indicate whether the sample is representative. Provide a rationale for the study sample chosen. For studies involving existing datasets, please describe the dataset and source.* |
| Sampling strategy | *Describe the sampling procedure (e.g. random, snowball, stratified, convenience). Describe the statistical methods that were used to predetermine sample size OR if no sample-size calculation was performed, describe how sample sizes were chosen and provide a rationale for why these sample sizes are sufficient. For qualitative data, please indicate whether data saturation was considered, and what criteria were used to decide that no further sampling was needed.* |
| Data collection | *Provide details about the data collection procedure, including the instruments or devices used to record the data (e.g. pen and paper, computer, eye tracker, video or audio equipment) whether anyone was present besides the participant(s) and the researcher, and whether the researcher was blind to experimental condition and/or the study hypothesis during data collection.* |
| Timing | *Indicate the start and stop dates of data collection. If there is a gap between collection periods, state the dates for each sample cohort.* |
| Data exclusions | *If no data were excluded from the analyses, state so OR if data were excluded, provide the exact number of exclusions and the rationale behind them, indicating whether exclusion criteria were pre-established.* |
| Non-participation | *State how many participants dropped out/declined participation and the reason(s) given OR provide response rate OR state that no participants dropped out/declined participation.* |
| Randomization | *If participants were not allocated into experimental groups, state so OR describe how participants were allocated to groups, and if allocation was not random, describe how covariates were controlled.* |

# Ecological, evolutionary & environmental sciences study design

All studies must disclose on these points even when the disclosure is negative.

| | |
|---|---|
| Study description | *Briefly describe the study. For quantitative data include treatment factors and interactions, design structure (e.g. factorial, nested, hierarchical), nature and number of experimental units and replicates.* |
| Research sample | *Describe the research sample (e.g. a group of tagged Passer domesticus, all Stenocereus thurberi within Organ Pipe Cactus National Monument), and provide a rationale for the sample choice. When relevant, describe the organism taxa, source, sex, age range and any manipulations. State what population the sample is meant to represent when applicable. For studies involving existing datasets, describe the data and its source.* |
| Sampling strategy | *Note the sampling procedure. Describe the statistical methods that were used to predetermine sample size OR if no sample-size calculation was performed, describe how sample sizes were chosen and provide a rationale for why these sample sizes are sufficient.* |
| Data collection | *Describe the data collection procedure, including who recorded the data and how.* |
| Timing and spatial scale | *Indicate the start and stop dates of data collection, noting the frequency and periodicity of sampling and providing a rationale for these choices. If there is a gap between collection periods, state the dates for each sample cohort. Specify the spatial scale from which the data are taken* |
| Data exclusions | *If no data were excluded from the analyses, state so OR if data were excluded, describe the exclusions and the rationale behind them, indicating whether exclusion criteria were pre-established.* |
| Reproducibility | *Describe the measures taken to verify the reproducibility of experimental findings. For each experiment, note whether any attempts to repeat the experiment failed OR state that all attempts to repeat the experiment were successful.* |
| Randomization | *Describe how samples/organisms/participants were allocated into groups. If allocation was not random, describe how covariates were controlled. If this is not relevant to your study, explain why.* |
| Blinding | *Describe the extent of blinding used during data acquisition and analysis. If blinding was not possible, describe why OR explain why blinding was not relevant to your study.* |

Did the study involve field work? ☐ Yes ☐ No

## Field work, collection and transport

| | |
|---|---|
| Field conditions | *Describe the study conditions for field work, providing relevant parameters (e.g. temperature, rainfall).* |
| Location | *State the location of the sampling or experiment, providing relevant parameters (e.g. latitude and longitude, elevation, water depth).* |
| Access & import/export | *Describe the efforts you have made to access habitats and to collect and import/export your samples in a responsible manner and in compliance with local, national and international laws, noting any permits that were obtained (give the name of the issuing authority, the date of issue, and any identifying information).* |
| Disturbance | *Describe any disturbance caused by the study and how it was minimized.* |

# Reporting for specific materials, systems and methods

We require information from authors about some types of materials, experimental systems and methods used in many studies. Here, indicate whether each material, system or method listed is relevant to your study. If you are not sure if a list item applies to your research, read the appropriate section before selecting a response.

### Materials & experimental systems

| n/a | Involved in the study | |
|---|---|---|
| ☐ | ☒ | Antibodies |
| ☐ | ☒ | Eukaryotic cell lines |
| ☒ | ☐ | Palaeontology and archaeology |
| ☐ | ☒ | Animals and other organisms |
| ☒ | ☐ | Clinical data |
| ☒ | ☐ | Dual use research of concern |
| ☒ | ☐ | Plants |

### Methods

| n/a | Involved in the study | |
|---|---|---|
| ☒ | ☐ | ChIP-seq |
| ☐ | ☒ | Flow cytometry |
| ☒ | ☐ | MRI-based neuroimaging |

## Antibodies

Antibodies used

1. APC-Cy7 Rat Anti-Mouse CD45(BD Pharmingen, Cat.557659; monoclonal; 1:100 for Flow Cyt)
2. PerCP-Cy5.5 Hamster Anti-Mouse CD3e(BD Pharmingen, Cat 551163; monoclonal; 1:100 for Flow Cyt)
3. FITC Rat Anti-Mouse CD8a(BD Pharmingen, Cat. 553030; monoclonal; 1:100 for Flow Cyt)
4. PE Anti-Mouse CD4 (Biolegend, Cat. 100511; monoclonal; 1:100 for Flow Cyt)
5. FITC Hamster Anti-Mouse CD11c(Biolegend, Cat. 117305; monoclonal; 1:100 for Flow Cyt)
6. PE-Cy7 Rat Anti-Mouse CD86(BD Pharmingen, Cat. 560582; monoclonal; 1:100 for Flow Cyt)
7. APC Rat anti-Mouse CD80 (BD Pharmingen, Cat. 560016; monoclonal; 1:100 for Flow Cyt)
8. Rabbit recombinant monoclonal Calreticulin antibody-conjugated to Alexa Flour 488(Abcam; Cat. ab196158; monoclonal; 1:400 for ICC/IF; Flow Cyt)
9. Alexa Flour 488-conjugated Goat Anti-Rabbit IgG (Servicebio; Cat. GB25303; polyclonal; 3:10 for IF; FC)
10. Cy3 conjugated Donkey Anti-Rabbit IgG (Servicebio; Cat. GB21403; polyclonal; 1:10 for IF; FC)
11. Recombinant Anti-CD3 antibody (Rabbit mAb) (Servicebio; Cat. GB150004; monoclonal; 1:1000 for IHC/IF)
12. Recombinant Anti-CD4 antibody (Rabbit mAb) (Servicebio; Cat. GB15064; monoclonal; 1:200 for IHC/IF)
13. Recombinant Anti-CD8 alpha antibody (Rabbit mAb) (Servicebio; Cat. GB15068; monoclonal; 1:400 for IHC/IF)
14. Anti-CD86 antibody (Abcam; Cat. ab270719; monoclonal; 1:200 for ICC/IF; IHC-P)
15. Anti-mannose receptor/CD206 Rabbit aAb (Servicebio; Cat. GB113497; polyclonal; 1:400 for IHC/IF)

Validation

Information regarding validation and application of the antibodies used can be found in the manufacturers' websites

## Eukaryotic cell lines

Policy information about cell lines and Sex and Gender in Research

| | |
|---|---|
| Cell line source(s) | A375(Cat. CL-0014), HepG3B(Cat. CL-0102), B16(Cat. CL-0319) cells were purchased from Procell system (Wuhan, China). VX2 (Cat. BFN60700420)cells were purchased from Shanghai BLUEFBIO Product. BMDC cells were acquired from C57B6 mice by a reported method (Nat. Immunol. 2008; 9(11): 1261-1269) |
| Authentication | A375, Hep3B, B16 and VX2 cell were morphologically confirmed according to the information provided by the cell-source center. STR analysis were performed in Supplementary Table 2-4. STR loci are amplified using fluorescently labeled PCR primers that flank the hypervariable regions. For VX2 cells, a certificate of analysis were performed from Shanghai BLUEFBIO Product. BMDCs cells were identified by immunstaining of biomarkers of CD11c. |

| | |
|---|---|
| Mycoplasma contamination | The cells were all negative in Mycoplasma test. |
| Commonly misidentified lines (See ICLAC register) | We double checked our cell lines in version 8.0 of ICLAC. There is no misidentified case report on these cells. |

## Palaeontology and Archaeology

| | |
|---|---|
| Specimen provenance | *Provide provenance information for specimens and describe permits that were obtained for the work (including the name of the issuing authority, the date of issue, and any identifying information). Permits should encompass collection and, where applicable, export.* |
| Specimen deposition | *Indicate where the specimens have been deposited to permit free access by other researchers.* |
| Dating methods | *If new dates are provided, describe how they were obtained (e.g. collection, storage, sample pretreatment and measurement), where they were obtained (i.e. lab name), the calibration program and the protocol for quality assurance OR state that no new dates are provided.* |

☐ Tick this box to confirm that the raw and calibrated dates are available in the paper or in Supplementary Information.

| | |
|---|---|
| Ethics oversight | *Identify the organization(s) that approved or provided guidance on the study protocol, OR state that no ethical approval or guidance was required and explain why not.* |

Note that full information on the approval of the study protocol must also be provided in the manuscript.

## Animals and other research organisms

Policy information about studies involving animals; ARRIVE guidelines recommended for reporting animal research, and Sex and Gender in Research

| | |
|---|---|
| Laboratory animals | Female C57B6 mice (6-8 weeks old, ~20g) were obtained from the Experimental Animal Center at Hangzhou Medical College, China. Female New Zealand White rabbits (6 months old, weighing between 2-2.5kg) were obtained from Qingdao Kangda Rabbit Co,. Ltd. (Qingdao, China). All animals were grown in an animal facility under filtered air conditions (22-25C), with a 12-hour light/dark cycle and relative humidity ranging from 40-70%, in plastice cages using sterilized wood shavings as beding.All animal experiments were strictly performed under the guidelines of the Chinese Council for Animal Care, approved by the Animal Care Committee of The Second Affiliated Hospital of Zhejiang University School of Medicine (ARIB-2023-1520). |
| Wild animals | No wild animals were used in this study. |
| Reporting on sex | The sex of the animals used in this study were Female mice and female rabbits,Age matched mice and rabbits were used. Sex was not considered in the study design. |
| Field-collected samples | No field-collected samples were used in this study. |
| Ethics oversight | All animal experiments were performed in accordance with the guidelines approved by the Animals Care Committee of the Laboratory Animals ar Zhejiang University ARIB-2023-1520 |

Note that full information on the approval of the study protocol must also be provided in the manuscript.

## Clinical data

Policy information about clinical studies

All manuscripts should comply with the ICMJE guidelines for publication of clinical research and a completed CONSORT checklist must be included with all submissions.

| | |
|---|---|
| Clinical trial registration | *Provide the trial registration number from ClinicalTrials.gov or an equivalent agency.* |
| Study protocol | *Note where the full trial protocol can be accessed OR if not available, explain why.* |
| Data collection | *Describe the settings and locales of data collection, noting the time periods of recruitment and data collection.* |
| Outcomes | *Describe how you pre-defined primary and secondary outcome measures and how you assessed these measures.* |

## Dual use research of concern

Policy information about dual use research of concern

### Hazards

Could the accidental, deliberate or reckless misuse of agents or technologies generated in the work, or the application of information presented in the manuscript, pose a threat to:

| No | Yes | |
|---|---|---|
| ☐ | ☐ | Public health |
| ☐ | ☐ | National security |
| ☐ | ☐ | Crops and/or livestock |
| ☐ | ☐ | Ecosystems |
| ☐ | ☐ | Any other significant area |

### Experiments of concern

Does the work involve any of these experiments of concern:

| No | Yes | |
|---|---|---|
| ☐ | ☐ | Demonstrate how to render a vaccine ineffective |
| ☐ | ☐ | Confer resistance to therapeutically useful antibiotics or antiviral agents |
| ☐ | ☐ | Enhance the virulence of a pathogen or render a nonpathogen virulent |
| ☐ | ☐ | Increase transmissibility of a pathogen |
| ☐ | ☐ | Alter the host range of a pathogen |
| ☐ | ☐ | Enable evasion of diagnostic/detection modalities |
| ☐ | ☐ | Enable the weaponization of a biological agent or toxin |
| ☐ | ☐ | Any other potentially harmful combination of experiments and agents |

## Plants

| | |
|---|---|
| Seed stocks | This study does not involve Plants |
| Novel plant genotypes | N/A |
| Authentication | N/A |

## ChIP-seq

### Data deposition

☐ Confirm that both raw and final processed data have been deposited in a public database such as GEO.

☐ Confirm that you have deposited or provided access to graph files (e.g. BED files) for the called peaks.

| | |
|---|---|
| Data access links<br>*May remain private before publication.* | *For "Initial submission" or "Revised version" documents, provide reviewer access links. For your "Final submission" document, provide a link to the deposited data.* |
| Files in database submission | *Provide a list of all files available in the database submission.* |
| Genome browser session<br>(e.g. UCSC) | *Provide a link to an anonymized genome browser session for "Initial submission" and "Revised version" documents only, to enable peer review. Write "no longer applicable" for "Final submission" documents.* |

### Methodology

| | |
|---|---|
| Replicates | *Describe the experimental replicates, specifying number, type and replicate agreement.* |
| Sequencing depth | *Describe the sequencing depth for each experiment, providing the total number of reads, uniquely mapped reads, length of reads and whether they were paired- or single-end.* |
| Antibodies | *Describe the antibodies used for the ChIP-seq experiments; as applicable, provide supplier name, catalog number, clone name, and lot number.* |
| Peak calling parameters | *Specify the command line program and parameters used for read mapping and peak calling, including the ChIP, control and index files used.* |
| Data quality | *Describe the methods used to ensure data quality in full detail, including how many peaks are at FDR 5% and above 5-fold enrichment.* |

Software | *Describe the software used to collect and analyze the ChIP-seq data. For custom code that has been deposited into a community repository, provide accession details.*

# Flow Cytometry

## Plots

Confirm that:

- [x] The axis labels state the marker and fluorochrome used (e.g. CD4-FITC).
- [x] The axis scales are clearly visible. Include numbers along axes only for bottom left plot of group (a 'group' is an analysis of identical markers).
- [x] All plots are contour plots with outliers or pseudocolor plots.
- [x] A numerical value for number of cells or percentage (with statistics) is provided.

## Methodology

Sample preparation

1. To expolre in vitro DC maturation, Bone Marrow(BM) Derived Dendritic Cells (BMDCs) were produced from the BM of 8-week-old C57B6 mice.The procedure involved cutting the hind legs of the mouse, removing the attached tissue, and sterilizing the femur and tibia by soaking them in 75% aclcohol for 5-10 seconds. the bones were the washed with PBS, and both ends were cut off. A syringe needle was inserted into the bone to flush the BM in the medium. The BM extract was carefully agitated, and cells were separated by centrifugation at 400g for 5 mins. The cell pellet was dissolved, and the cell density regulated to 1000000/mL prior to being introduced into RPMI-1640 medum containing recombinant mouse GM-CSF (20ng/mL) and IL-4 (10 ng/mL). The meidum was exchanged every 72 hours, and immature dendritic cells were harvested on day 8. Subsequently, 1000000 immature DC cells were co-cultured with pre-treated B16 cells for 24 hours. the maturation of DC cells was then assessed by staining with anti-CD11c-FITC, anti-CD80-PE, and anti-CD86-APC antibodies and analyzed using flow cytometry.
2. For intratumoral inflitration of T lumphocytes, To prepare a tumor single-cell suspension, tumors were excised on day 3 post-treatment, sectioned into small pieces, and enzymatically digested in DMEM containing 1mg/mL collagenase IV (C8160, Solarbio, China) and 0.2 mg/mL DNase I (D8071, Solarbio, China) for 45 mins. Isolated cells were stained with anti-CD45-APC-Cy7, anti-CD3-PerCP-Cy5.5, anti-CD8-FITC, and anti-CD4-PE antibodies for 30 mins and subsequently analyzed by flow cytometry.
3. For CRT expression assay. A375 and B16 cells were plated in 6-well plates at 500000 cells per well and incubated for 24 hours. After treatments by PB@MCs and other groups for 6 hours, cells were rinsed with cold PBS (1mL) and fixed in 0.25% paraformaldehyde(1mL) for 10 mins,  then incubated with Alexa Fluor 488-conjugated CRT primary antibody for 30 mins before undergoing flow cytometry.
4. To measure IFN-r levels, whold blood was collected from 8-week-old C57B6 mice by eyeball blood collection on day 3 after different treatment, Blood was centrifuged at 3000rpm for 5 mins, and the serum was extracted. Levels of IFN-r were quantified using an ELISA kit as per the provided insructions.
5. B16, VX2, Hep3B and A375 cells were cultured in 6-transwell plated at 500000 cells/well, after different treatment for 8 hours, cells were harvested and stained using an Annexin V-FITC/DAPI apoptosis kit, then analyzed via flow cytometry.

Instrument | The instrument for data collection is FACSCanto II BD.

Software | Data analysis was carried out using FlowJo software 10.6.2 (Tree Star Inc.)

Cell population abundance | At least 10,000 total events were acquired for all analyses.

Gating strategy | In general, cells were first gated by FSC-A/SSC-A to exclude debris, then FSC-A/FSC-H to exclude doublets followed by specific antibody stain ( e.g. CD4 etc).

- [x] Tick this box to confirm that a figure exemplifying the gating strategy is provided in the Supplementary Information.

# Magnetic resonance imaging

## Experimental design

Design type | *Indicate task or resting state; event-related or block design.*

Design specifications | *Specify the number of blocks, trials or experimental units per session and/or subject, and specify the length of each trial or block (if trials are blocked) and interval between trials.*

Behavioral performance measures | *State number and/or type of variables recorded (e.g. correct button press, response time) and what statistics were used to establish that the subjects were performing the task as expected (e.g. mean, range, and/or standard deviation across subjects).*

## Acquisition

Imaging type(s) | *Specify: functional, structural, diffusion, perfusion.*

Field strength | *Specify in Tesla*

Sequence & imaging parameters | *Specify the pulse sequence type (gradient echo, spin echo, etc.), imaging type (EPI, spiral, etc.), field of view, matrix size, slice thickness, orientation and TE/TR/flip angle.*

Area of acquisition | *State whether a whole brain scan was used OR define the area of acquisition, describing how the region was determined.*

Diffusion MRI ☐ Used ☐ Not used

## Preprocessing

Preprocessing software | *Provide detail on software version and revision number and on specific parameters (model/functions, brain extraction, segmentation, smoothing kernel size, etc.).*

Normalization | *If data were normalized/standardized, describe the approach(es): specify linear or non-linear and define image types used for transformation OR indicate that data were not normalized and explain rationale for lack of normalization.*

Normalization template | *Describe the template used for normalization/transformation, specifying subject space or group standardized space (e.g. original Talairach, MNI305, ICBM152) OR indicate that the data were not normalized.*

Noise and artifact removal | *Describe your procedure(s) for artifact and structured noise removal, specifying motion parameters, tissue signals and physiological signals (heart rate, respiration).*

Volume censoring | *Define your software and/or method and criteria for volume censoring, and state the extent of such censoring.*

## Statistical modeling & inference

Model type and settings | *Specify type (mass univariate, multivariate, RSA, predictive, etc.) and describe essential details of the model at the first and second levels (e.g. fixed, random or mixed effects; drift or auto-correlation).*

Effect(s) tested | *Define precise effect in terms of the task or stimulus conditions instead of psychological concepts and indicate whether ANOVA or factorial designs were used.*

Specify type of analysis: ☐ Whole brain ☐ ROI-based ☐ Both

Statistic type for inference | *Specify voxel-wise or cluster-wise and report all relevant parameters for cluster-wise methods.*

(See Eklund et al. 2016)

Correction | *Describe the type of correction and how it is obtained for multiple comparisons (e.g. FWE, FDR, permutation or Monte Carlo).*

## Models & analysis

| n/a | Involved in the study |
|---|---|
| ☐ | ☐ Functional and/or effective connectivity |
| ☐ | ☐ Graph analysis |
| ☐ | ☐ Multivariate modeling or predictive analysis |

Functional and/or effective connectivity | *Report the measures of dependence used and the model details (e.g. Pearson correlation, partial correlation, mutual information).*

Graph analysis | *Report the dependent variable and connectivity measure, specifying weighted graph or binarized graph, subject- or group-level, and the global and/or node summaries used (e.g. clustering coefficient, efficiency, etc.).*

Multivariate modeling and predictive analysis | *Specify independent variables, features extraction and dimension reduction, model, training and evaluation metrics.*
